# Cell fusion in the brain: two cells forward, one cell back

**DOI:** 10.1007/s00401-014-1303-1

**Published:** 2014-06-05

**Authors:** Kevin Kemp, Alastair Wilkins, Neil Scolding

**Affiliations:** Multiple Sclerosis and Stem Cell Group, School of Clinical Sciences, University of Bristol, Neuroscience Office, Learning and Research Building, Southmead Hospital, Bristol, BS10 5NB UK

**Keywords:** Fusion, Purkinje cells, Heterokaryon, Inflammation, Cerebellum, Bone marrow

## Abstract

Adult stem cell populations, notably those which reside in the bone marrow, have been shown to contribute to several neuronal cell types in the rodent and human brain. The observation that circulating bone marrow cells can migrate into the central nervous system and fuse with, in particular, cerebellar Purkinje cells has suggested, at least in part, a potential mechanism behind this process. Experimentally, the incidence of cell fusion in the brain is enhanced with age, radiation exposure, inflammation, chemotherapeutic drugs and even selective damage to the neurons themselves. The presence of cell fusion, shown by detection of increased bi-nucleated neurons, has also been described in a variety of human central nervous system diseases, including both multiple sclerosis and Alzheimer’s disease. Accumulating evidence is therefore raising new questions into the biological significance of cell fusion, with the possibility that it represents an important means of cell-mediated neuroprotection or rescue of highly complex neurons that cannot be replaced in adult life. Here, we discuss the evidence behind this phenomenon in the rodent and human brain, with a focus on the subsequent research investigating the physiological mechanisms of cell fusion underlying this process. We also highlight how these studies offer new insights into endogenous neuronal repair, opening new exciting avenues for potential therapeutic interventions against neurodegeneration and brain injury.

## Introduction

The inherent cellular complexity and intricate neural circuitry of the central nervous system (CNS) presents significant theoretical challenges to strategies aimed at rescuing injured and dying neurons in neurodegenerative conditions. The observation that circulating bone marrow (BM) cells can migrate into the CNS and fuse with (in particular) cerebellar Purkinje cells offers a promising and feasible mechanism to overcome some of these potential difficulties.

Adult stem cells have been defined as undifferentiated multi-potent cells with the capacity to renew and to differentiate into all the major mature cell types in the organ they reside. Thus, neural stem cells differentiate down multiple neuronal lineages to form mature neuronal cells and other stem cell types will also follow this pattern: hematopoietic stem cells, for example, differentiate into the various cells of the hematopoietic system. This paradigm of stem cell plasticity in the CNS was, however, challenged by controversial studies using BM chimeric mice reporting that stem cell populations from distinct lineages, which typically reside in the BM, could form multiple neuronal cell types in the brain [6, 27]. Consequently, these findings sparked much interest into the mechanisms underlying this phenomenon of cell plasticity. Was this unconventional trans-differentiation of BM-derived cells across germ layers? Or were there alternative explanations—could fusion of BM-derived cells with mature neurons explain the observations? Over the past few years, studies attempting to address these questions have offered new insights into neuronal repair, and opened exciting new avenues for potential therapeutic interventions.

### Bone into brain?

Conventional theories of stem cell plasticity in the CNS were questioned at the beginning of the millennium, when studies by both Mezey et al. [27] and Brazelton et al. [6] suggested that BM-derived cells could enter the brain and trans-differentiate into cells with a neuronal-specific phenotype. Donor BM stem cells, identified using the male Y chromosome in female recipients or genetically tagged with the green fluorescent protein (GFP), were transplanted peripherally into either lethally irradiated or mutant PU.1 null mice (PU.1 is a member of the ETS family of transcription factors expressed exclusively by cells of the hematopoietic lineage; a mutation in the PU.1 gene renders these mice incapable of developing cells of myeloid and lymphoid lineages and consequently, without a BM transplant, they die 48 h after birth.) Subsequently cells of BM origin expressing neuronal-specific antigens including NeuN, NF-H and BIII-tubulin were all found (as early as 1 month post-transplant) in modest numbers in CNS anatomical sites including the olfactory bulb, cerebral cortex, hypothalamus, hippocampus, amygdala, periaqueductal gray and striatum.

A year later both Priller and Nakano [30, 35], like Brazelton et al., utilised BM chimerism in rodents, in which GFP-tagged BM stem cells were transplanted into lethally irradiated mice. Again shortly after transplant, BM-derived cells were found in the brain, but negligible numbers of GFP cells co-expressed neuronal markers, such as NeuN and NSE, and none showed morphological characteristics of neurons. Interestingly, however, and wholly unforeseen, was the observation that, 12 months post-transplant, up to 0.1 % of Purkinje cells within the cerebellum expressed GFP: no other brain region contained GFP-labelled neurons. These cells, with a typical Purkinje cell morphology and expressing Calbindin-D28K, displayed extensive GFP expression localised in the perikaryon, axon and dendritic tree. They also expressed markers implying neurotransmitter synthesis, such as GABA-synthesising enzyme and glutamic acid decarboxylase; and they made multiple synaptic contacts, suggesting the GFP-expressing Purkinje cells were at least functional. At this time it was proposed, although rare, the formation of these GFP-expressing Purkinje cells was the result of neural differentiation of infiltrating GFP-marked blood-borne cells.

### Cell fusion in the brain

**Fig. 1:**
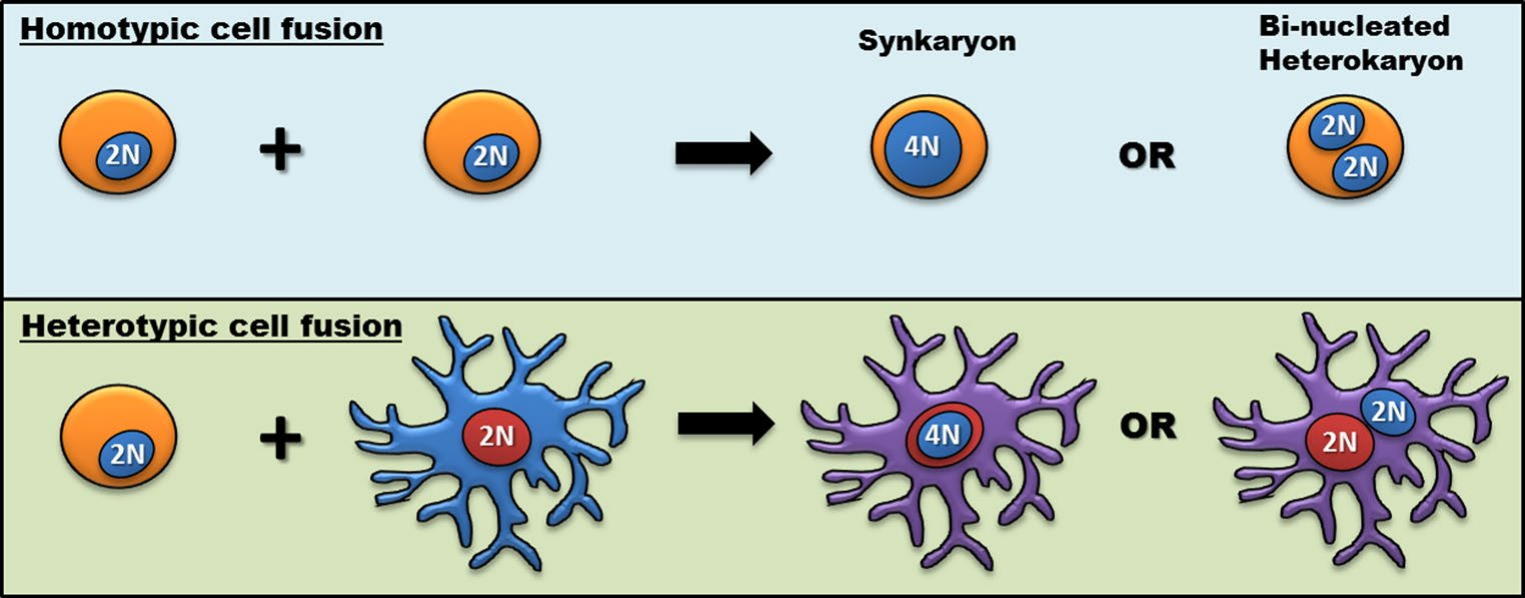
The different forms of cell fusion. Cell fusion can occur between two or more cells of the same lineage (homotypic) or between genetically different cell types (heterotypic) without subsequent chromosomal loss. Cellular fusion can also result in either the absence or amalgamation of nuclear material to form multi-nucleate (heterokaryon) and mono-nucleate (synkaryon) cells, respectively

**Fig. 2:**
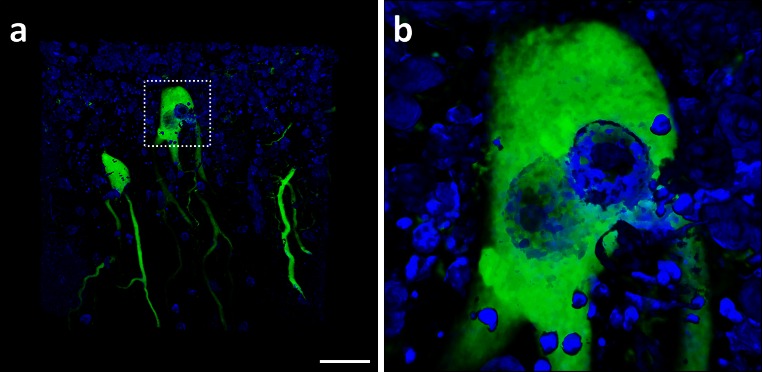
A bi-nucleate Purkinje cell within the human cerebellum. A 3D confocal image of cells within the human cerebellum immunofluorescently labelled with the Purkinje cell-specific marker Calbindin-D28K (*green*) and DAPI nuclear stain (*blue*). The hatched area in **a** represents the higher magnified image (**b**) (*scale bar* 25 μm)

This unexpected BM-derived cell trans-differentiation into neurons was quickly questioned; the alternative of heterotypic cell fusion and the subsequent transfer of ‘donor’ genetic material to form bi-nucleate heterokaryons (see Fig. 1) was put forward to account for the appearance of donor-derived Purkinje cells [1, 45]. Bi-nucleated neurons have been described in a variety of human CNS pathologies, including Alzheimer’s disease [50], neuro-Behcet’s disease [39], multiple sclerosis [18], Kuru [19] and spino-olivo-ponto-cerebello-nigral atrophy [16]. Historically, as far back as 1939, studies had documented bi-nucleate Purkinje cells in humans [2] (see Fig. 2). Several decades later, reports had also shown that the DNA content of a small percentage of Purkinje cells could be hyperdiploid, with many displaying twice the amount of nuclear DNA of somatic cells [21, 22, 25, 26]. We now know this corroborates quite nicely with what is seen as a result of cellular fusion and the formation of bi-nucleate heterokaryons. However, at the time controversy surrounded these facts and a critical appraisal of the techniques used in those studies claiming a tetraploid DNA content of Purkinje cells strongly contradicted their findings and they were therefore seemingly dismissed [24].

### Bone marrow-derived cells in the human brain

Human studies then followed, using fluorescent in situ hybridization (FISH) to identify the Y chromosome in post-mortem brain tissue from females who had received sex-mismatched BM transplants for either haematological malignancies or genetic deficiencies of the immune system. Mezey et al. [28] in 2003 showed in four patients tested (survival between 2 and 10 months post-transplant) that BM cells contributed to neuronal populations in the CNS, particularly in both the neocortex and hippocampal regions. The frequency of these Y chromosome-positive cells was 2–7 cells per 10,000 neurons. It was also noted that a plethora of non-neuronal cells, thought to be oligodendrocytes, astrocytes, microglia, endothelial, meningeal and ependymal cells, were also found all bearing the Y chromosome. A year later, a similar study by Cogle et al. examined the hippocampus from three female sex-mismatched BM transplant recipients. Again both microglia and astrocytes were found to bear the Y chromosome (with up to 2 % being labelled). Furthermore, and maybe more surprisingly, in one patient 6 years post-transplant 1 % of all neurons within the hippocampus were found to contain donor-derived Y chromosomes. Conversely, no Y chromosome-labelled neurons were found in the remaining two patients. This was considered likely due to the short periods of donor engraftment (both patients dying within 2 months post-transplant) [8]. Interestingly, the patient with significant neuronal engraftment was the only individual who had received a whole BM transplant. The two remaining patients had received peripheral blood stem cell harvests, highlighting, although highly speculatively, possible distinctions between different donor stem cell sources in their capacity to engraft within the CNS. The bone marrow population is indeed a heterogeneous one. There are many stem cell sub-populations present, including both haematopoietic and mesenchymal precursors, all of which have shown to have the ability to contribute to the Purkinje cell population. Yet, no studies to date have comprehensively compared the fusogenic capabilities of different donor cell sources or populations, with the vast majority of transplantation studies using whole BM preparations.

In these human studies described, large numbers of cells were analysed; however, no signs of donor-derived polyploidic cells were evident and thus fusion was considered unlikely and trans-differentiation of BM cells was proposed to be the more likely explanation for BM-derived cells in the brain. However, Weimann et al. [44] in comparable studies concentrating on the cerebellum, found that BM-derived stem cells contributed to Purkinje cells in adult women who had received male BM transplants. Again using FISH to detect BM-derived cells, the total frequency of Purkinje cells harbouring the donor Y chromosome was approximately 0.1 % in patients 3–15 months post-transplant. The novel observation of this study was that two Purkinje cells were found with more than a diploid sex chromosome composition (both a XXY and XXX phenotype was found), raising the tentative prospect that BM-derived cells donate genetic material to Purkinje cells through fusion events between these two distinct cell types. (Note: when interpreting the presence of Y chromosome-positive cells in the females after male BM transplantation, the possible confounding factor regarding feto-maternal chimerism and the transfer of cells from the male foetus to its mother must be considered [41]. The child-bearing status of the female subjects was not reported in these studies, therefore the observation of Y chromosomes in the brain being a result of feto-maternal chimerism cannot be ruled out.)

### Purkinje cells

Purkinje cells are a class of GABAergic neurons located in the cerebellar cortex and are some of the largest and most complex neurons in the human brain. Their axons are the sole outputs from the cerebellar cortex and the extensive dendritic network from a single Purkinje cell can receive synaptic inputs from as much as 200,000 parallel fibres [43]. They are therefore critical for normal cerebellar function, and as such, an essential part of the motor system regulating muscle tone and movement [20]. Traditionally Purkinje cells, in both rodent and humans, are mono-nucleate diploid cells that lack the ability to undergo cell division [24, 29, 49]. They are generated only during early brain development and in contrast to other neurons there is no evidence of their generation after birth [44].

### Bone marrow-derived cells fuse with Purkinje cells in the rodent brain

**Fig. 3:**
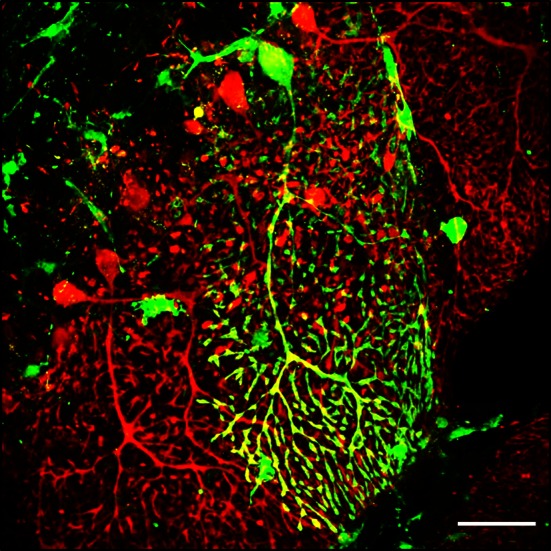
A GFP-labelled bone marrow-derived Purkinje cell. A single GFP-positive Purkinje cell found within the cerebellum of a BM chimeric mouse (expressing GFP-tagged bone marrow) with experimental autoimmune encephalomyelitis (EAE). The image represents fusion between a GFP-labelled (*green*) bone marrow cell and a Calbindin-D28K positive Purkinje cell (*red*). Several GFP-positive glial cells can also be observed within the image. GFP-expressing bone marrow chimeras were produced through transplantation of GFP-tagged bone marrow stem cells into lethally irradiated mice (*scale bar* 50 μm)

The studies described above demonstrated unquestionably that BM-derived cells, although at very low levels, can cross the blood–brain barrier and contribute to the neuronal architecture of the CNS including Purkinje cells within the cerebellum (see Fig. 3). Nevertheless, it remained unclear whether the underlying mechanism was trans-differentiation and generation of neuronal cells de novo or BM-derived cell fusion with the existing neuronal cells, or both.

In 2003, using Cre/lox recombination to detect fusion events, Alvarez-Dolado et al. [1] neatly demonstrated that BM-derived cells fused with Purkinje cells in the cerebellum (in addition to cardiomyocytes and hepatocytes) and in the process donated nuclear genetic material to the recipient neuronal cell. [Cre/lox recombination is an important technique that has been extensively used in cell fusion studies to conditionally turn on or off gene expression in specific cell types or tissues. Using conventional recombination strategies a transgenic strain of mice is developed in which a ‘target’ of interest is engineered to be flanked by two Lox P sites (in this specific case the target was an intervening stop sequence between the promoter and the coding region of the LacZ transgene). A second strain of mice is also engineered to carry a transgene for the Cre-recombinase gene with a tissue-specific or ubiquitous promoter. When Cre-expressing cells fuse with cells containing Lox P sites from another, the Cre enzyme excises the Lox P flanked target DNA. In this specific study, the transplantation of Cre-expressing donor cells and their subsequent fusion with recipient Purkinje cells containing Lox P sites resulted in the expression of LacZ which was in-turn visualised using X-gal labelling.] No less importantly, electron microscopy showed these cells to contain two nuclei; these nuclei being clearly distinct: one being that of a typical ‘Purkinje cell-like morphology, another being more spherical in shape with multiple nucleoli.

In the same year, Weimann et al. [45] also addressed the fusion/trans-differentiation conundrum using GFP-positive sex-mis-matched BM transplants in mice. GFP-positive Purkinje cells were found for well over a year post-transplant, with the number of these GFP-positive cells increasing in a linear fashion over the experimental period. No other neurons in the cerebellum expressed GFP. Furthermore, using detailed confocal microscopy, unexpectedly these GFP-positive cells were shown to contain two nuclei, with one of these nuclei containing the Y chromosome. This indeed presented, for the first time, compelling evidence that cells from the BM could fuse spontaneously with Purkinje cells in the cerebellum to form stable, non-dividing, bi-nucleate, chromosomally balanced heterokaryons. Large numbers of heterokaryons had one large ‘Purkinje-like’ nucleus with dispersed chromatin, and one smaller nucleus with a dense chromatin structure; the remaining cells contained two identical nuclei, both large with dispersed chromatin. Over time the proportion of the latter cells (containing two ‘Purkinje-like’ nuclei) increased considerably, suggesting once a BM-derived cell (with a compact nucleus) fuses with a Purkinje cell, its nucleus is reprogrammed and acquires the characteristics of a Purkinje cell. This hypothesis was further supported by the observation that GFP-positive Purkinje cells lacked expression of the hematopoietic markers CD11b, F4/80 and CD45 in mice after transplanting BM cells expressing GFP under the Purkinje cell-specific promoter L7-*pcp*-2. This proved that BM cells once fused with the existing Purkinje cell population were reprogrammed to express, in a genetically dominant fashion, Purkinje cell-specific genes.

### Is fusion an experimental artefact?

The discovery that BM-derived Purkinje cells resulted from fusion events, led to several studies over the next few years attempting to further understand this phenomenon. In both humans and rodents, without additional pathology such as inflammation, Purkinje cell fusion appeared to occur at very low levels, though the frequency of fusion events was thought to increase with age [23, 45, 46]. It was, however, debated whether many of these studies were conducted under physiological conditions as the patients or animals were subjected to highly invasive regimens involving either irradiation or chemotherapeutic drugs to remove the host haematopoietic system. Bi-nucleated Purkinje cells are found in aged (un-manipulated) mice, though the donor nucleus is of an unknown cellular origin [23]. Without question, direct radiation exposure to the cerebellum dramatically increases the incidence of fusion between BM-derived cells and Purkinje cells [12, 46]. Additionally, both chemotherapeutic BM-conditioning drugs alone and selective damage to Purkinje cells (for example using intraventricular injections of propidium iodide) increase fusion events [23]. All of these procedures are likely to result in increased permeability of the blood–brain barrier and/or increased levels of inflammation within the CNS [32, 42].

To address this problem in rodents, parabiosis experiments resulting in formation of blood chimerism were devised, thus negating the requirement for irradiation or chemotherapeutic drugs [15] (both experimental procedures proposed as prime candidates to cause fusion artefact). Parabiosis generates GFP blood chimerism through the surgical joining of two mice, one being the recipient wild-type mouse and the other a donor mouse ubiquitously expressing GFP. The parabiotic mice develop a common anastomosed circulatory system leading to blood chimerism within a week of surgery. ‘Wild-type’ parabiont mice display heterotypic GFP-positive Purkinje cells consistently within 20–26 weeks after surgery [15], providing evidence that irradiation and chemotherapeutic drugs were not required for fusion to occur (see also below).

Arguably the most stringent study conducted to date to elucidate whether the phenomenon of cell fusion occurs under true physiological conditions was carried out by Nern and colleagues in 2009 [31]. Using the Cre/Lox transgenic mouse model system to irreversibly label cells of the haematopoietic lineage, they were able to positively detect endogenous haematopoietic contribution to tissues, which was restricted to Purkinje cells in brain tissue, in the absence of both irradiation or chemoablation. The frequency of potentially fused cells in these animals was comparable to those found in previous studies involving bone marrow transplantation. Furthermore, under these experimental conditions, using techniques including FISH, confocal and electron microscopy, fused cells were shown to be mono-nucleate and showing no signs of polyploidy [31].

### Inflammation

It remains the case that little is known about the regulation or physiological significance of Purkinje cell fusion. If cell fusion plays a physiologically significant role in Purkinje cell development and/or survival, could the frequency of fusion be modified experimentally? In 2008, two studies by Johansson et al. and Nygren et al. [15, 33] sought to address this conundrum using in vivo models to characterise the conditions under which heterotypic cell fusion arises and whether conditions exist to increase their frequency. In studies by Johansson et al. chronic inflammation (through induction of either autoimmune encephalomyelitis or idiopathic ulcerative dermatitis) caused substantial increases in fusion between BM-derived cells and Purkinje cells to form, in all cases, bi-nucleate heterokaryons. The increases in fusion events observed were 10- to 100-fold higher than that reported in previous studies [1, 44, 45]. They also elegantly showed that fusion between BM-derived cells and Purkinje cells led to reprogramming of the donor nucleus, resulting in expression of Purkinje cell-specific genes. Furthermore, the cellular progeny of a single transplanted hematopoietic stem cell (SP, Lin^−^, Sca1^+^c-kit^+^) could fuse with Purkinje cells, giving tentative clues to the cellular origins of the fusing ‘donor’ cells. Nygren et al. [33] also studied heterotypic fusion of BM-derived cells with Purkinje cells alongside other cell types, including cardiomyocytes, muscle fibres and hepatocytes. Here they also showed, using Cre-Lox recombination technology that not only cells of a myeloid lineage could fuse with Purkinje cells, but cells of the lymphoid lineage were equally able to undergo cell fusion. In addition, they suggested that the haematopoietic contribution to Purkinje cells may have a non-myeloid and distinct origin from that of bone marrow-derived microglia cells [33]. Fusion events were also amplified in response to irradiation injury; a phenomenon interestingly inhibited through the administration of the anti-inflammatory corticosteroid prednisolone. This provided further compelling evidence that inflammatory cues allow significant numbers of fused cells to be observed experimentally.

Whether an increase in fusion events is the direct result of inflammation-induced migration and infiltration of cells, linked to increased permeability of the blood–brain barrier is unknown. Studies within our laboratory have shown in vitro that fusion events may be, in part, mediated as a secondary effect of cell infiltration and/or soluble factors including TNF-alpha and IFN-gamma, released in the initiation and amplification of the local immune response in the CNS post-tissue injury [17]. Nern et al. [31] also observe an increase in the frequency of Purkinje cell fusion events (without the generation of bi-nucleated cells) after intrathecal delivery of lipopolysaccharide (LPS) and corresponding microglia infiltration in the cerebellum. However, to confuse matters, although a different approach to trace BM contribution to Purkinje neurons was used, experiments involving intraperitoneal injections of LPS into mice producing large influxes of macrophage/microglia in the cerebellum lack subsequent increases in heterokaryon formation [15] (LPS is a potent endotoxin capable of stimulating the hosts’ innate immune response, resulting in the activation of macrophages and microglia, leading to the formation and release of a large spectrum of inflammatory mediators [37]). Furthermore, in parabionts with dermatitis, large numbers of heterokaryons were found, yet microglial/macrophage were rarely found within the cerebellum of these animals [15]. As a consequence, no correlation between infiltration of microglia/macrophages and the formation of heterokaryons in the cerebellum seems to be evident: circulating soluble factors may be more important.

In humans, taking advantage of the inflammatory nature of multiple sclerosis, studies again within our laboratories have shown for the first time a disease-related increase in Purkinje cell fusion and heterokaryon formation. In this study, heterokaryon formation, although very rare, was observed to take place in control subjects with no inflammatory or neurodegenerative neuropathological changes at post-mortem, but the frequency of this event was considerably increased in patients with multiple sclerosis with approximately 0.4 % of Purkinje cells being bi-nucleate heterokaryons. Furthermore, in agreement with the above observations from rodent studies, no correlation between heterokaryon formation and active or chronic cerebellar inflammation was evident, nor was heterokaryon formation preferentially at lesion sites [18].

### Is fusion a stable or a transient process?

It must be emphasised that experimentally, during a single snapshot in time, Purkinje cell fusion and heterokaryon formation is indeed rare. If fusion between BM cells and Purkinje cells to form stable heterokaryons constitutes a regenerative process to introduce healthy nuclei or functional genes into aged or degenerating cells, then it may be expected that greater numbers should be observed in humans, especially in older subjects or instances where injury to the cerebellum occurs. Alternatively, if the bi-nucleate state of fused cells is indeed a transient ‘snapshot’ of the pathological process, the amount of fusion taking place could be significantly underestimated. As described above, we have found rare instances where bi-nucleated Purkinje cells are evident in control patients with no neuropathological changes at post-mortem (and had not received any prior BM transplantation). However, other human studies have not found any bi-nucleate or and polyploidic Purkinje cells in non-transplanted individuals [31].

In mice, the frequency of fusion events does increase with time, suggesting fusion results in the formation of stable reprogrammed heterokaryons [23, 45, 46]. Nevertheless, this may simply be a result of an increased requirement for Purkinje cell fusion/regeneration attributable to age-related processes. Conversely, in aged GFP chimeric mice, bi-nucleated Purkinje cells have been found to largely outnumber their GFP-positive Purkinje cell counterparts. One explanation is that the fused nucleus is quickly inactivated [23]. Experiments designed to address whether GFP-labelled heterokaryons persist long-term in the cerebellum have been undertaken, relying on the removal of the GFP chimerism in these models to prevent further GFP-labelled heterokaryons to be formed [15, 23]. These studies do suggest that heterokaryons persist for long periods of time, some studies showing for up to 7 months, as heterokaryon frequency does not significantly drop after reversing GFP chimerism (through removal of a GFP-expressing parabiont or undertaking a second BM transplant of non-GFP-expressing cells). However, a major caveat to these studies is that GFP-positive cells were, although in much lower numbers, still present in the circulation, thus the generation of new GFP-expressing Purkinje cells de novo cannot be entirely ruled out.

The intriguing studies by Nern et al. [31] using the Cre/Lox transgenic mouse model system observed that hematopoietic genetic contribution to Purkinje cells could occur. However, as described above, unlike previous studies, they reported that these Purkinje cells contained only a single nucleus. They proposed that nuclear and/or protein transfer between hematopoietic cells and Purkinje cells, under these physiological conditions, occurred through transient fusion or intercellular vesicular transport mechanisms. The vesicular transfer of DNA, mRNA and even organelles from BM-derived cells to various different tissues has indeed been previously reported [36] and may be an important method of cellular communication and/or tissue repair.

### Fusion in genetic models of neurodegeneration

The frequency of Purkinje cell fusion events increases with age, radiation exposure, inflammation, chemotherapeutic drugs and even selective damage to Purkinje cells. This raises the possibility that fusion represents a means of cell-mediated neuroprotection or rescue of highly differentiated cell types which cannot be replaced in adults [5, 40]. Purkinje cells are generated only during early brain development and in contrast to other neurons there is no evidence of their generation after birth [44]. Consequently, significant loss in Purkinje cell numbers as a result of toxic, autoimmune, genetic and neurodegenerative insults generally leads to irreversible reduced motor function and ataxia. Clinically, harnessing fusion events leading to preservation in Purkinje cell numbers could therefore have valuable implications in a wide range of patients with cerebellar degeneration and disease. Liver repopulation with functional BM-derived hepatocytes in genetic liver disease, a result of fusion between donor BM-derived cells and host hepatocytes, has already been shown in animal models, thereby providing strong proof of principle for targeted organ regeneration through exploiting cell fusion events [47].

Using genetic models of Purkinje cell degeneration, researchers have explored the potential of using cellular fusion as a means of Purkinje cell rescue. For instance, in transgenic mice expressing mutated superoxide dismutase 1 (SOD1), an animal model of amyotrophic lateral sclerosis (ALS; a motor neuron degenerative disease), transplantation of GFP-expressing wild-type BM cells led to GFP-positive neurons (not exclusively Purkinje cells), including some containing two nuclei, detected in both the brain and spinal cord [9]. In murine Niemann–Pick Type C1 disease (NP-C; a lysosomal storage disease disorder in humans characterised by progressive ataxia, cerebellar atrophy, psychomotor deterioration, extrapyramidal deficits and dementia), where mice undergo a characteristic pattern of severe Purkinje cell loss, increasing from lobe I of the anterior zone to lobe VII of the posterior cerebellar vermis, transplantation of BM stem cells directly into the cerebellum alleviates Purkinje cell degeneration [3]. Initial studies by Bae et al. [3] revealed that Purkinje cells 4 weeks post-transplant express donor BM-derived GFP alongside endogenous cellular proteins, with many of these cells again containing two nuclei. Intriguingly they reported that Purkinje cell fusion was increased in the NP-C1 brain when compared to wild-type mice, signifying that the neurodegenerative microenvironment in NP-C1 mice appeared to augment this phenomenon. Further studies also provided a significant breakthrough in understanding the functionality of fused Purkinje cells. Using whole-cell patch clamp recordings they showed for the first time that fusion between BM-derived cells and existing Purkinje cells led to the formation of electrically active neurons with functional synaptic formation within a neurodegenerative cerebellar environment [4].

Several known murine genetic mutations also lead to impairments in motor and cerebellar function, such as the Purkinje cell degeneration (PCD) mutation characterised by reduced expression of the *Agtpbp1* gene. Homozygous mice display a rapid and dramatic loss of Purkinje cells by 20 days of age causing severe ataxia [10]. In this phenotype, a lack of disease-related Purkinje cell fusion is seen after irradiation/transplantation of GFP-expressing BM cells, possibly a result of the timing at which BM transplantation occurred and the extremely rapid and severe irreversible onset of Purkinje cell degeneration in this model [10]. (However, it is reported in this model that BM-derived cells did contribute to olfactory bulb neurons, but through differentiation, not fusion.) Conversely, mice heterozygous for the (PCD) mutation exhibit a slow but significant age-dependent decrease in Purkinje cell number, and importantly, without any detectable inflammation or reactive gliosis. In these heterozygous mice, following transplantation of GFP-expressing BM cells, fusion with Purkinje cells is observed. More notably, the frequency of fusion and heterokaryon formation is increased when compared to transplanted age-matched controls, thus signifying the somewhat mild degenerative environment can stimulate fusion events [11].

Understanding the circumstances in which cell fusion and heterokaryon formation occur may lead to techniques to manipulate these mechanisms therapeutically, providing the opportunity to introduce functional/healthy ‘donor’ genetic material that may boost Purkinje cell survival. A study by Chen et al. [7] set out to prove this concept in a mouse model of spinocerebellar ataxia 1 (SCA1) (*Sca1* ^*154Q/2Q*^ mice) (SCA1 is an autosomal dominant disorder caused by the expansion of a CAG tri-nucleotide repeat expansion in the coding region of the *Sca1* gene, resulting in neurodegeneration of specific neuronal populations in both the CNS and PNS including severe Purkinje cells loss). Using transplantation of genetically modified male BM cells carrying the *SCA1* gene into female irradiated *Sca1* ^*154Q/2Q*^ mice, they showed that bi-nucleated Purkinje cells heterokaryons containing the Y chromosome were detected post-transplant. Furthermore, these cells expressed the SCA1 modifier genes in vivo, presenting for the first time, evidence that cell fusion could be utilised as a mode of neuroprotective gene therapy in disorders involving Purkinje cell degeneration.

### Final thoughts

Degeneration of the cerebellum, and particularly Purkinje cells therein, occurs in many neurological disorders, including multiple sclerosis, spinocerebellar ataxias, stroke, metabolic disturbances (such as chronic alcoholism), cancer and direct trauma. Heterotypic cell fusion, with the potential to protect and rescue neuronal cells and restore homeostatic balance during neurodegeneration, is a phenomenon that may be amenable to therapeutic manipulation. There is an air of elegance in the concept of fusion as a rescue process by which blood cells migrate into the CNS and donate genetic material to injured highly complex cell types that otherwise cannot be replaced in adults through classical modes of trans-differentiation. With this in mind, it could be hypothesised that cell fusion would appear as an extremely efficient evolutionary mechanism of cell rescue when compared to a complete cell replacement.

In contrast to its seemingly simple nature, membrane fusion between two different cells is mediated by a number of distinct and structurally unrelated membrane fusion-molecules. These molecules mediate the initial recognition of the membranes that are destined for fusion and pull the membranes close together to destabilise the lipid/water interface and to initiate the intricate mixing of the lipids merging the two lipid bilayers to become one [14]. On completion of membrane fusion, we know from chromosome analysis and gene expression that a nucleus is donated into the recipient cell. It would be intriguing to speculate whether other organelles are simultaneously donated? Transfer of such cellular components could be key to cellular repair in a number of neurodegenerative conditions. Outside the brain, BM stem cells can certainly protect tissues through transferring mitochondria to vulnerable cells via the formation of nanotubes and microvesicles [13]. Furthermore, exosomes from BM cells can mediate transfer of microRNAs to neuronal cells, regulating their gene expression, leading to functional recovery in rodent models of stroke [48].

What happens to both the donated and endogenous nuclei post-fusion is largely unknown. Morphological evidence of nuclear reprogramming is evident as the donated nucleus seems to acquire the characteristics of the host Purkinje cell [45]. Genes derived from the donated nucleus are clearly expressed within its host, demonstrated in chimeric animals where fused Purkinje cells fluoresce green through translation of the GFP transgene. Moreover, post-fusion, donated nuclei are also reprogrammed to express Purkinje cell-specific genes [15, 45]. How these nuclear changes are made still needs to be investigated. Clues are given in vitro, where fusion between somatic and progenitor cells can lead to changes in cell signalling, DNA methylation and potency [34]. Furthermore, recent in vivo studies have shown that fusion and reprogramming of retinal neurons is mediated through Wnt/β-catenin signalling pathways [38].

**Fig. 4:**
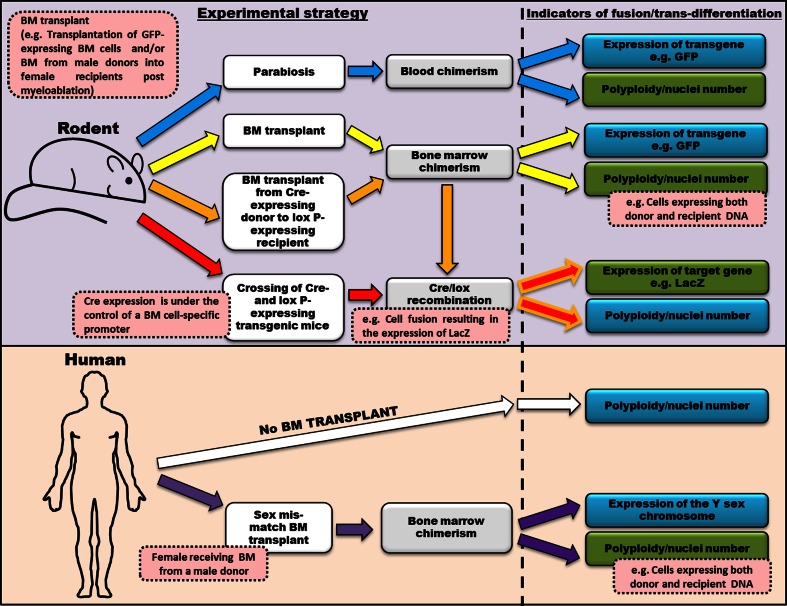
A schematic representation of the different experimental strategies used to study the integration of bone marrow cells in the brain of both rodents and humans. The image depicts different experimental strategies (each alternative method indicated using a *coloured arrow*) reported to investigate how BM-derived cells contribute (through both fusion and trans-differentiation) to neurons in the brain. Indicators of BM-derived cell fusion/trans-differentiation in the brain highlighted in *green boxes* are those of which can be used to detect true fusion events. Examples of studies reporting these experiential strategies are: *blue arrows* [15], *yellow arrows* [45], *orange arrows* [1], *red arrows* [31], *white arrows* [18] and *purple arrows* [44]

It is clear that non-neuronal cell types of BM origin, including both macro- and micro-glia, are found in the brain [8, 28]. To date, there has been no suggestion that these cells have arisen through mechanisms of fusion, with trans-differentiation of primitive BM-derived precursor cells being the only hypothesis put forward for their existence. The reasons why only Purkinje cells, out of the huge plethora of neuronal (and non-neuronal) sub-types in the CNS, appear to have the ability to specifically fuse with BM-derived cells is of much interest. It may be that Purkinje cells have an embryological propensity to polyploidy or it may simply be related to their large size and complex structure [21, 25]. Although many studies using different experimental strategies (see Fig. 4) have described BM-derived cell fusion in the cerebellum as specific to Purkinje cells, comparably intense analysis of other CNS areas, such as the hippocampus, olfactory bulb, neocortex and spinal cord, where significant numbers of BM-derived neuron-like cells have been found, is (arguably) still awaited. Whether their presence is due to fusion or trans-differentiation processes still needs to be clarified. Taking into consideration the heterogeneous nature of the BM cell population, it is possible that BM-derived cells contribute to neurons through a number of different mechanisms (including both that of fusion and trans-differentiation). It must be emphasised that failure to identify either bi-nucleated neurons or the expression of endogenous transgenes does certainly not exclude fusion. Fusion could simply result in the subsequent loss of donor or recipient chromosomes. Furthermore, from our own experience, methods of identifying bi-nucleate cells are, at best, challenging and are very likely to yield an underestimate of the true frequency of fusion events.

It is tempting to speculate that understanding the mechanisms underlying cell fusion may lead to techniques to increase the frequency of fusion events and to deliver ‘healthy donor’ cells and/or genes to degenerating neuronal cells. Given this potential solution to repairing neurons in adult life, harnessing fusion could be clinically valuable to a vast number of neurological diseases: a very small percentage difference in cell survival could be of disproportionate functional benefit in diseases where pharmacological approaches have proved, thus far, wholly ineffective.

## References

[1] Alvarez-Dolado M, Pardal R, Garcia-Verdugo JM, Fike JR, Lee HO, Pfeffer K, Lois C, Morrison SJ, Alvarez-Buylla A (2003). Fusion of bone-marrow-derived cells with Purkinje neurons, cardiomyocytes and hepatocytes. Nature.

[2] Andrew W (1939). Origin and significance of binucleate Purkinje cells in man. Arch Pathol.

[3] Bae JS, Furuya S, Shinoda Y, Endo S, Schuchman EH, Hirabayashi Y, Jin HK (2005). Neurodegeneration augments the ability of bone marrow-derived mesenchymal stem cells to fuse with Purkinje neurons in Niemann-Pick type C mice. Hum Gene Ther.

[4] Bae JS, Han HS, Youn DH, Carter JE, Modo M, Schuchman EH, Jin HK (2007). Bone marrow-derived mesenchymal stem cells promote neuronal networks with functional synaptic transmission after transplantation into mice with neurodegeneration. Stem Cells.

[5] Blau HM (2002). A twist of fate. Nature.

[6] Brazelton TR, Rossi FM, Keshet GI, Blau HM (2000). From marrow to brain: expression of neuronal phenotypes in adult mice. Science.

[7] Chen KA, Cruz PE, Lanuto DJ, Flotte TR, Borchelt DR, Srivastava A, Zhang J, Steindler DA, Zheng T (2011). Cellular fusion for gene delivery to SCA1 affected Purkinje neurons. Mol Cell Neurosci.

[8] Cogle CR, Yachnis AT, Laywell ED, Zander DS, Wingard JR, Steindler DA, Scott EW (2004). Bone marrow transdifferentiation in brain after transplantation: a retrospective study. Lancet.

[9] Corti S, Locatelli F, Donadoni C, Guglieri M, Papadimitriou D, Strazzer S, Del Bo R, Comi GP (2004). Wild-type bone marrow cells ameliorate the phenotype of SOD1-G93A ALS mice and contribute to CNS, heart and skeletal muscle tissues. Brain J Neurol.

[10] Diaz D, Recio JS, Baltanas FC, Gomez C, Weruaga E, Alonso JR (2011). Long-lasting changes in the anatomy of the olfactory bulb after ionizing irradiation and bone marrow transplantation. Neuroscience.

[11] Diaz D, Recio JS, Weruaga E, Alonso JR (2012). Mild cerebellar neurodegeneration of aged heterozygous PCD mice increases cell fusion of Purkinje and bone marrow-derived cells. Cell Transplant.

[12] Espejel S, Romero R, Alvarez-Buylla A (2009). Radiation damage increases Purkinje neuron heterokaryons in neonatal cerebellum. Ann Neurol.

[13] Islam MN, Das SR, Emin MT, Wei M, Sun L, Westphalen K, Rowlands DJ, Quadri SK, Bhattacharya S, Bhattacharya J (2012). Mitochondrial transfer from bone-marrow-derived stromal cells to pulmonary alveoli protects against acute lung injury. Nat Med.

[14] Jahn R, Lang T, Sudhof TC (2003). Membr fusion. Cell.

[15] Johansson CB, Youssef S, Koleckar K, Holbrook C, Doyonnas R, Corbel SY, Steinman L, Rossi FM, Blau HM (2008). Extensive fusion of haematopoietic cells with Purkinje neurons in response to chronic inflammation. Nat Cell Biol.

[16] Kaiya H (1974). Spino-olivo-ponto-cerebello-nigral atrophy with Lewy bodies and binucleated nerve cells: a case report. Acta Neuropathol.

[17] Kemp K, Gordon D, Wraith DC, Mallam E, Hartfield E, Uney J, Wilkins A, Scolding N (2011). Fusion between human mesenchymal stem cells and rodent cerebellar Purkinje cells. Neuropathol Appl Neurobiol.

[18] Kemp K, Gray E, Wilkins A, Scolding N (2012). Purkinje cell fusion and binucleate heterokaryon formation in multiple sclerosis cerebellum. Brain J Neurol.

[19] Klatzo I, Gajdusek DC, Zigas V (1959). Pathology of Kuru. Lab Investig J Tech Methods Pathol.

[20] Kozorovitskiy Y, Gould E (2003). Stem cell fusion in the brain. Nat Cell Biol.

[21] Lapham LW (1968). Tetraploid DNA content of Purkinje neurons of human cerebellar cortex. Science.

[22] Lentz RD, Lapham LW (1969). A quantitative cytochemical study of the DNA content of neurons of rat cerebellar cortex. J Neurochem.

[23] Magrassi L, Grimaldi P, Ibatici A, Corselli M, Ciardelli L, Castello S, Podesta M, Frassoni F, Rossi F (2007). Induction and survival of binucleated Purkinje neurons by selective damage and aging. J Neurosci.

[24] Mann DM, Yates PO, Barton CM (1978). The DNA content of Purkinje cells in mammals. J Comp Neurol.

[25] Mares V, Lodin Z, Sacha J (1973). A cytochemical and autoradiographic study of nuclear DNA in mouse Purkinje cells. Brain Res.

[26] Mares V, van der Ploeg M (1980). Cytophotometric re-investigation of DNA content in Purkinje cells of the rat cerebellum. Histochemistry.

[27] Mezey E, Chandross KJ, Harta G, Maki RA, McKercher SR (2000). Turning blood into brain: cells bearing neuronal antigens generated in vivo from bone marrow. Science.

[28] Mezey E, Key S, Vogelsang G, Szalayova I, Lange GD, Crain B (2003). Transplanted bone marrow generates new neurons in human brains. Proc Natl Acad Sci USA.

[29] Miyata M, Miyata H, Mikoshiba K, Ohama E (1999). Development of Purkinje cells in humans: an immunohistochemical study using a monoclonal antibody against the inositol 1,4,5-triphosphate type 1 receptor (IP3R1). Acta Neuropathol.

[30] Nakano K, Migita M, Mochizuki H, Shimada T (2001). Differentiation of transplanted bone marrow cells in the adult mouse brain. Transplantation.

[31] Nern C, Wolff I, Macas J, von Randow J, Scharenberg C, Priller J, Momma S (2009). Fusion of hematopoietic cells with Purkinje neurons does not lead to stable heterokaryon formation under noninvasive conditions. J Neurosci.

[32] Neuwelt EA, Glasberg M, Frenkel E, Barnett P (1983). Neurotoxicity of chemotherapeutic agents after blood-brain barrier modification: neuropathological studies. Ann Neurol.

[33] Nygren JM, Liuba K, Breitbach M, Stott S, Thoren L, Roell W, Geisen C, Sasse P, Kirik D, Bjorklund A, Nerlov C, Fleischmann BK, Jovinge S, Jacobsen SE (2008). Myeloid and lymphoid contribution to non-haematopoietic lineages through irradiation-induced heterotypic cell fusion. Nat Cell Biol.

[34] Patel M, Yang S (2010). Advances in reprogramming somatic cells to induced pluripotent stem cells. Stem Cell Rev.

[35] Priller J, Persons DA, Klett FF, Kempermann G, Kreutzberg GW, Dirnagl U (2001). Neogenesis of cerebellar Purkinje neurons from gene-marked bone marrow cells in vivo. J Cell Biol.

[36] Prockop DJ (2012). Mitochondria to the rescue. Nat Med.

[37] Rivest S (2009). Regulation of innate immune responses in the brain. Nat Rev Immunol.

[38] Sanges D, Romo N, Simonte G, Di Vicino U, Tahoces AD, Fernandez E, Cosma MP (2013). Wnt/beta-catenin signaling triggers neuron reprogramming and regeneration in the mouse retina. Cell Rep.

[39] Shintaku M, Kaneda D (2012). Binucleated neurons in the pontine nuclei in neuro-Behcet’s disease: a study of 3 autopsy cases. Clin Neuropathol.

[40] Singec I, Snyder EY (2008). Inflammation as a matchmaker: revisiting cell fusion. Nat Cell Biol.

[41] Tan KH, Zeng XX, Sasajala P, Yeo A, Udolph G (2011). Fetomaternal microchimerism: some answers and many new questions. Chimerism.

[42] Trnovec T, Kallay Z, Bezek S (1990). Effects of ionizing radiation on the blood brain barrier permeability to pharmacologically active substances. Int J Radiat Oncol Biol Phys.

[43] Tyrrell T, Willshaw D (1992). Cerebellar cortex: its simulation and the relevance of Marr’s theory. Philos Trans R Soc Lond B Biol Sci.

[44] Weimann JM, Charlton CA, Brazelton TR, Hackman RC, Blau HM (2003). Contribution of transplanted bone marrow cells to Purkinje neurons in human adult brains. Proc Natl Acad Sci USA.

[45] Weimann JM, Johansson CB, Trejo A, Blau HM (2003). Stable reprogrammed heterokaryons form spontaneously in Purkinje neurons after bone marrow transplant. Nat Cell Biol.

[46] Wiersema A, Dijk F, Dontje B, van der Want JJ, de Haan G (2007). Cerebellar heterokaryon formation increases with age and after irradiation. Stem Cell Res.

[47] Willenbring H, Bailey AS, Foster M, Akkari Y, Dorrell C, Olson S, Finegold M, Fleming WH, Grompe M (2004). Myelomonocytic cells are sufficient for therapeutic cell fusion in liver. Nat Med.

[48] Xin H, Li Y, Liu Z, Wang X, Shang X, Cui Y, Gang Zhang Z, Chopp M (2013). Mir-133b promotes neural plasticity and functional recovery after treatment of stroke with multipotent mesenchymal stromal cells in rats via transfer of exosome-enriched extracellular particles. Stem Cells.

[49] Zecevic N, Rakic P (1976). Differentiation of Purkinje cells and their relationship to other components of developing cerebellar cortex in man. J Comp Neurol.

[50] Zhu X, Siedlak SL, Wang Y, Perry G, Castellani RJ, Cohen ML, Smith MA (2008). Neuronal binucleation in Alzheimer disease hippocampus. Neuropathol Appl Neurobiol.

